# Supplementary material for: Potential of an Interorgan Network Mediated by Toxic Advanced Glycation End-Products in a Rat Model
Source: Nutrients. 2020 Dec 29;13(1):80. doi: 10.3390/nu13010080 (PMC7823945; doi:10.3390/nu13010080)
Supplement: Supplementary file 1 [file nutrients-13-00080-s001.pdf]

Table S1. Body weight, water intake, and urine volume.

	Control	HFCS
**Final body weight (g)**	343.8 ± 19.3	356.1 ± 21.0
**Water intake (mL)**	36.5 ± 2.3	67.5 ± 4.9*
**Urine volume (mL)**	23.8 ± 8.3	63.3 ± 6.5*

Control; control group, HFCS; high-fructose corn syrup (HFCS) group.

Table S2. Urinary examination.

	Control	HFCS
**pH**	7.2 ± 0.3	6.1 ± 0.1*
**CRE (mg/day)**	8.3 ± 1.0	8.1 ± 0.6
**UA (mg/day)**	2.0 ± 1.5	2.1 ± 0.2
**Ca (mg/day)**	1.5 ± 1.2	2.3 ± 0.3
**Oxalate (mg/day)**	0.5 ± 0.1	0.6 ± 0.1
**Citrate (mg/day)**	11.6 ± 6.3	15.5 ± 1.3
**8-OHdG (ng/24 h/g)**	0.41 ± 0.1	0.43 ± 0.1

Data is shown as mean ± standard deviation (n = 10 in each group). Control; control group, HFCS; high-fructose corn syrup (HFCS) group, UV; urine volume, CRE; creatinine, UA; uric acid, Ca; calcium, 8-OHdG: 8-hydroxy-2'-deoxyguanosine, measured value/24 h urine volume/body weight (ng/24 h/g). Data are shown as mean ± standard deviation (n = 10 in each group). P values are based on Mann Whitney-U test. *$P < 0.05$ versus the control group.

Table S3. Blood examination.

	Control	HFCS
**CRE (mg/dL)**	0.3 ± 0.0	0.3 ± 0.0
**UA (mg/dL)**	1.5 ± 0.5	1.9 ± 0.5
**TG (mg/dL)**	75.4 ± 35.4	137.0 ± 33.9*
**LDL-C (mg/dL)**	7.7 ± 1.9	8.8 ± 2.2
**GLU (mg/dL)**	138.8 ± 30.0	132.8 ± 32.2
**HbA1c (%)**	5.03 ± 0.3	5.21 ± 0.2*

Data is shown as mean ± standard deviation (n = 10 in each group). Control; control group, HFCS; high-fructose corn syrup (HFCS) group, BUN; blood urea nitrogen, CRE; creatinine, UA; uric acid, TG; triglyceride, LDL-C; low-density lipoprotein cholesterol, GLU; glucose, HbA1c; glycated haemoglobin A1c. P values are based on Mann Whitney-U test. *$P < 0.05$ versus the control group.

Figure S1.

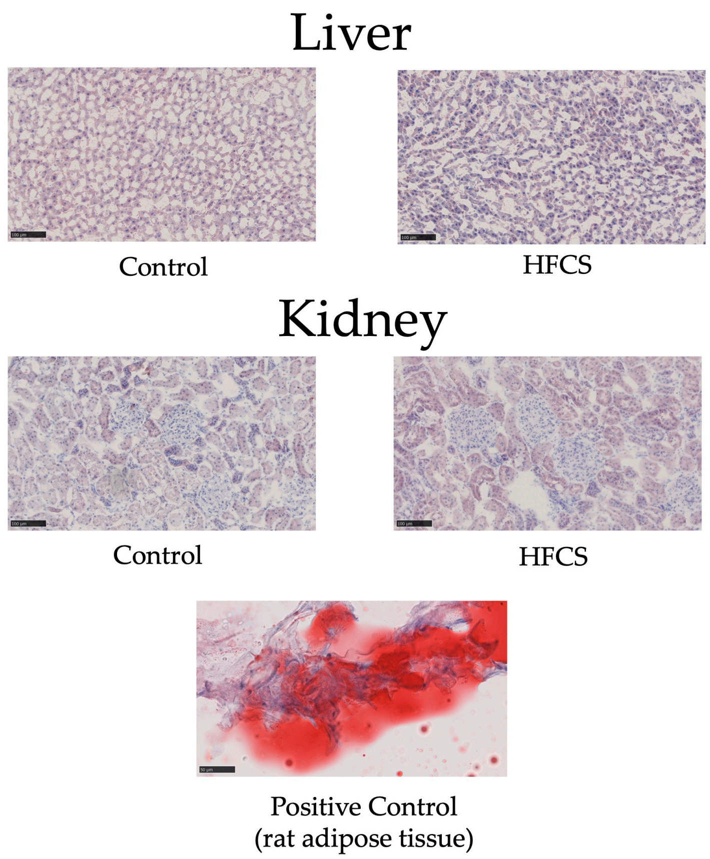

Figure S1. Oil Red O staining of the Liver and the Kidney. The scale bar represents 50μm.

Figure S2.

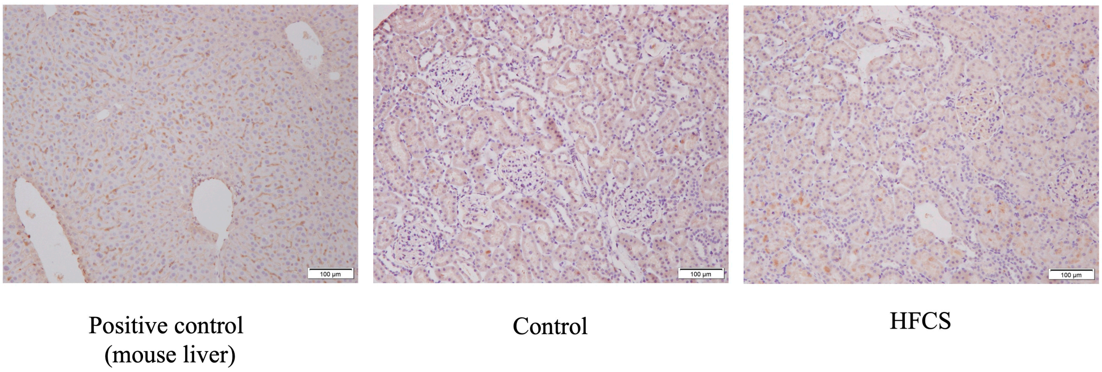

Figure S2. Immunostaining of CD68 in the kidney of paraffin sections. The scale bar represents 100 μm.

Figure S3.

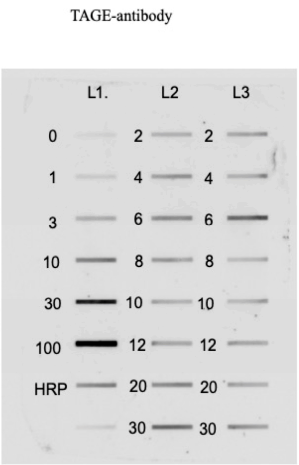

Figure S3. Intracellular toxic advanced glycation end-products (TAGE) in Wister rat kidneys were analysed using slot blot analysis. Cell lysates (2.0, 4.0, 6.0, 8.0, 10, 12, 20, and 30 μg of protein/lane) were blotted onto a polyvinylidene difluoride (PVDF) membrane. The quantity of TAGE was calculated based on a calibration curve for TAGE-bovine serum albumin (BSA). Proteins on the PVDF membranes were probed with the immunopurified anti-TAGE-antibody. L1: TAGE-BSA (0, 1, 3, 10, 30, and 100 ng/lane) and horseradish peroxidase (HRP)-linked molecular weight marker were blotted on the PVDF membrane. L2 and L3: Cell aaalysates (2.0, 4.0, 6.0, 8.0, 10, 12. 20, and 30 μg of protein/lane) were blotted onto the PVDF membrane.

Figure S4.

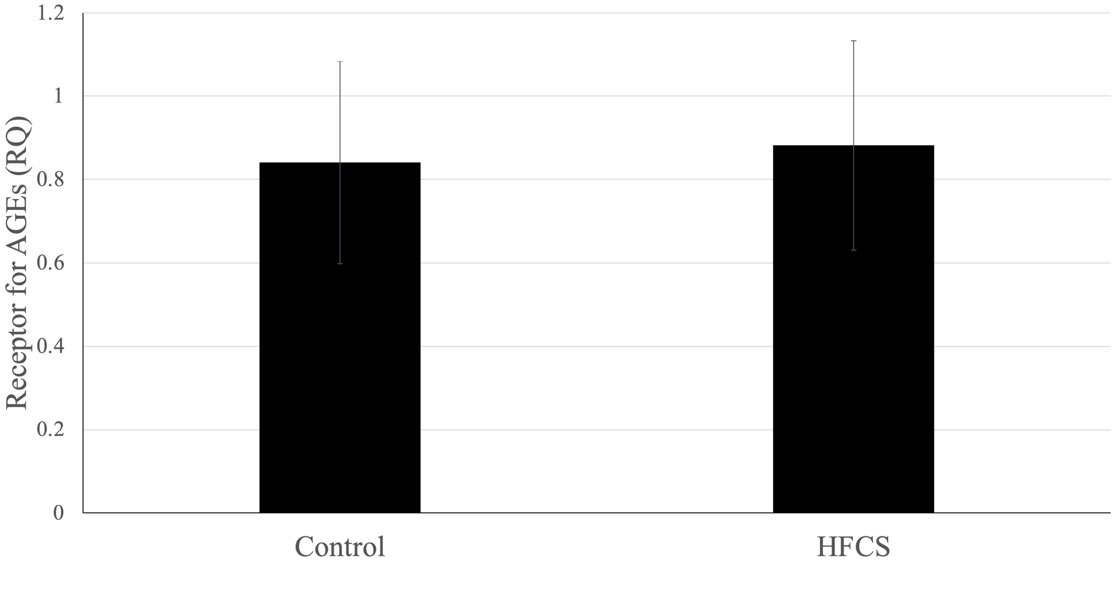

Figure S4. Gene expression of *receptor for AGEs* (*RAGE*) in the kidneys. Gene expression of *RAGE* was analysed with real-time polymerase chain reaction (RT-PCR). Normalised gene expression levels are shown as a ratio between the mean value for the target gene and that for β actin in

each sample (n = 10 in each group). P values were based on Mann Whitney-U test. RQ, relative quantification.

Figure S5.

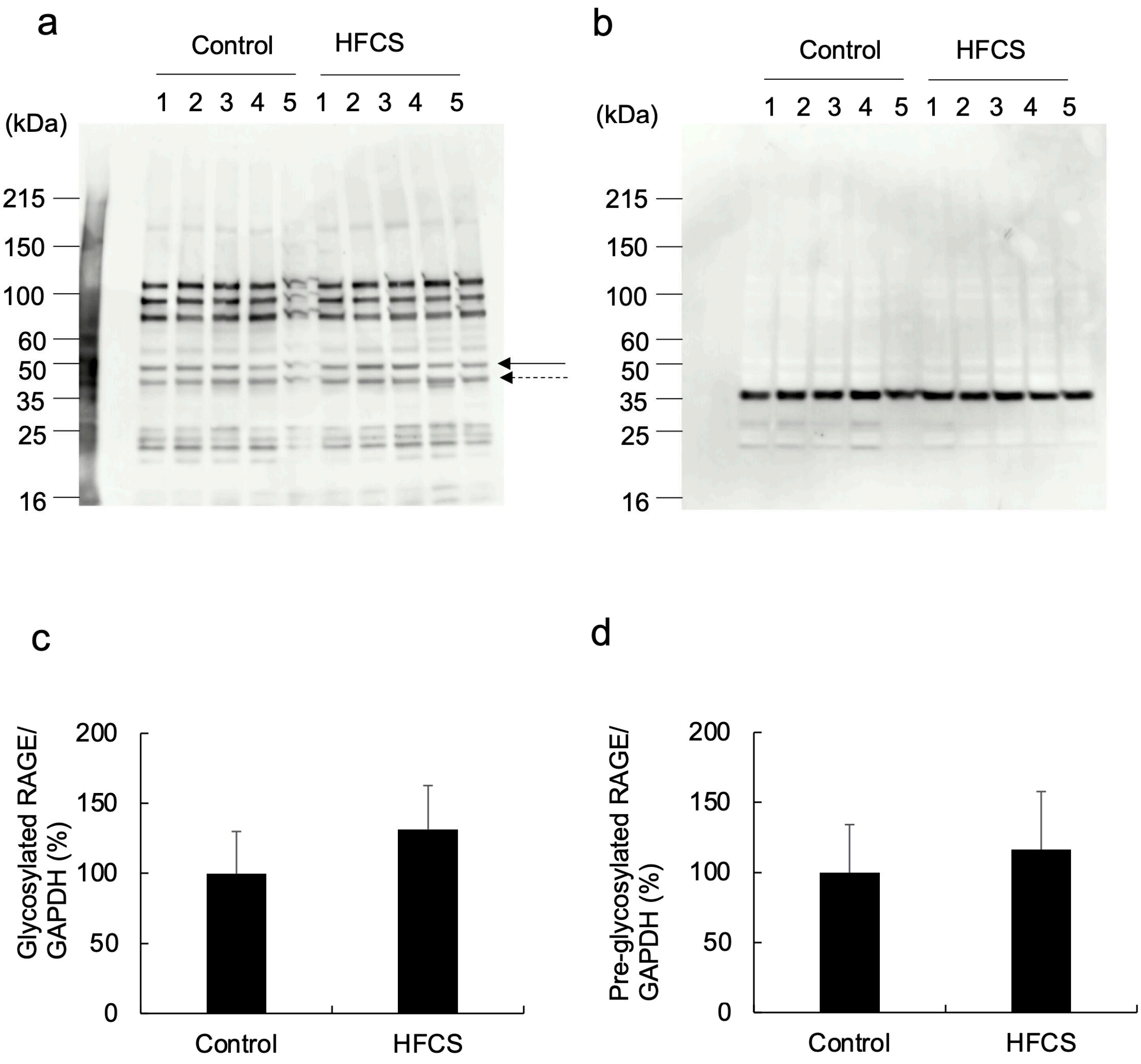

Figure S5. Expression of RAGE in the kidney. Control; control group (n = 5), HFCS; HFCS group (n = 5). The numbers 1-5 indicate each rat in the control or HFCS group. Tissue lysates (30 μg of protein/lane) were loaded on 4-15% gradient polyacrylamide gels. Proteins on polyvinylidene

difluoride (PVDF) membranes were probed with anti-RAGE and anti-GAPDH antibodies. (**a**) The band of RAGE was analyzed with Western blotting (WB). The solid arrow indicates the band of the glycosylated RAGE. The broken arrow indicates the band of the pre-glycosylated RAGE. (**b**) The band of GAPDH was analyzed with WB. (**c**) The expression of the glycosylated RAGE in the control and HFCS groups was normalized with GAPDH. (**d**) The expression of the pre-glycosylated RAGE in the control and HFCS groups was normalized with GAPDH. (**c**, **d**) Data are presented as mean ± SD (*n* = 5). *P* values are based on Mann Whitney-U test.

Figure S6.

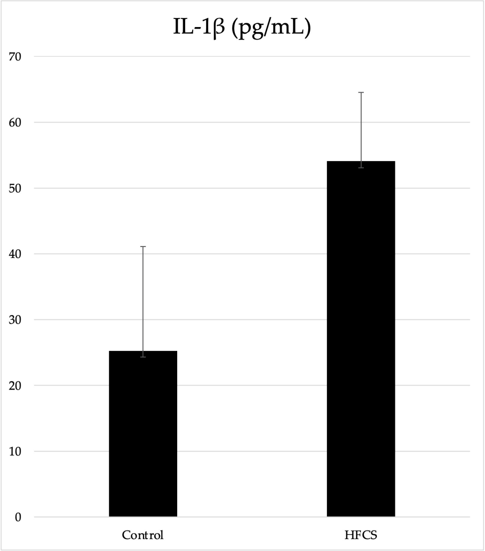

Figure S6. IL-1β in serum from control and HFCS group rats obtained from animal experiments.
P values are based on Mann Whitney-U test.
